# Evaluating the Impact of Direct, Direct Video, and Indirect Video Laryngoscopy Training on the Proficiency of Medical Students in Performing Direct Laryngoscopy: A High-Fidelity Manikin-Based Assessment

**DOI:** 10.7759/cureus.70984

**Published:** 2024-10-07

**Authors:** Sydney E Moriarty, Ishan R Perera, Mohammad Sabbagh, Matthew Yeckley, Paul Carpio, Arian Hoodfar, Alison LePera, Ramu Anandakrishnan, Taylor Daniels, Ryan Martin, Janella Looney, Kimberly Gittings, Watson Edwards, Frederic Rawlins, II

**Affiliations:** 1 Student Research Connect, Edward Via College of Osteopathic Medicine, Blacksburg, USA; 2 Emergency Medicine, Edward Via College of Osteopathic Medicine, Blacksburg, USA; 3 Biomedical Sciences, Edward Via College of Osteopathic Medicine, Blacksburg, USA; 4 Simulation and Educational Technology, Edward Via College of Osteopathic Medicine, Blacksburg, USA

**Keywords:** airway assessment, comparative techniques, glidescope, intubation injury, intubation training, laerdal platform, laryngoscopy techniques, manikin simulation, video intubation

## Abstract

Endotracheal intubation (ETI), a potentially lifesaving intervention employed frequently in the emergent setting, is a manual skill that improves with repetitive practice and high-quality feedback. Classically, ETI centered around Direct Laryngoscopy (DL); however, with the advent and recent availability of Indirect Video Laryngoscopy (IVL) and Direct Video Laryngoscopy (DVL), studies have demonstrated varying results on the benefit of Video Laryngoscopy (VL) in training. We hypothesize that a training program centered on DVL, allowing students to visualize the anatomy and simultaneously receive instructor feedback via a real-time video feed, will practically improve student performance in DL. Our study of first-year medical students from the Edward Via College of Osteopathic Medicine (n = 21) randomized participants to one of three cohorts: DL, IVL, and DVL in a manikin-based simulation laboratory evaluated on successful intubation, time to successful intubation, dental injury, Numeric Rating Scale (NRS) to assess the trainee’s perception of their performance and confidence level of performing intubation in a real-life scenario. Our results did not demonstrate a statistically significant difference between the three training modalities based on the outcomes assessed. Although IVL and DVL cohorts achieved 100% success following training, compared to 71% in the DL cohort, the results were not statistically significant. This is potentially due to our limited sample size, as our sample did not meet the calculated 162 participants for 80% power. These findings suggest that a larger sample size may be required to determine if there is a significant difference in outcomes for these training modalities.

## Introduction

Endotracheal intubation (ETI) is a common, potentially lifesaving, procedure frequently encountered in both emergency and surgical settings [1,2]. It is one of the most important manual skills in anesthesiology, subject to a learning curve like any other manual skill [3,4]. A study of medical students, respiratory therapy students, and paramedic students lacking prior ETI experience demonstrated a 47-attempt requirement to perform a "good" intubation if the subject inserts and lifts the laryngoscope successfully while asking for help when appropriate as based on statistical modeling [5]. Potential consequences of a failed intubation include oxygen desaturation, arrhythmias, cardiac arrest, brain damage, and mortality [6,7].

With the advent of new ETI tools, such as Video Laryngoscopy (VL), and acknowledging the role of operator skill, our investigation aimed to discern the most effective method of medical education for medical students acquiring this crucial skill through utilizing a high-fidelity manikin-based assessment. Specifically, we compared the effectiveness of training using Direct Laryngoscopy (DL) against Indirect Video Laryngoscopy (IVL) and Direct Video Laryngoscopy (DVL). Metrics used in our analysis included achievement of successful DL intubation on a manikin, time to successful intubation, dental injury, Numeric Rating Scale (NRS) to assess the trainee’s perception of their performance, and confidence level of performing intubation in a future real-life scenario.

Earlier research with medical student cohorts found that ETI training using DL first resulted in shorter mean intubation times compared to a cohort trained with VL [8]. In another study, novices were trained using a McGrath™ video laryngoscope (Medtronic, Minneapolis, MN), which can be used for both IVL and DVL [9]. Intubation times were significantly reduced in the DVL group compared to the IVL group, with higher difficulty reported in the IVL group [9]. The American Society of Anesthesiologists has defined a difficult airway as when "a conventionally trained anesthesiologist experiences difficulty with facemask ventilation of the upper airway, difficulty with tracheal intubation, or both" [10]. Factors contributing to the difficulty of an airway include the patient's status, clinical scenario requiring intubation, patient characteristics, medical history, and surgical history [11]. Previous work by Lim et al., in 2004, evaluating time to intubation in IVL-trained inexperienced medical students revealed the time to intubation was significantly shorter in cohorts utilizing IVL during difficult intubation [12]. In contrast, work done by Lim et al. in 2005 revealed experienced anesthesiologists took longer with IVL in comparison to DL during a simulated easy laryngoscopy [13]. During a simulated difficult laryngoscopy, anesthesiologists had faster times to successful intubation with IVL, though there was no difference in success rate when compared to DL [13].

Based on these findings, we hypothesized that utilizing DVL as the training modality would lead to improved outcomes in DL during a high-fidelity manikin simulation, compared to the IVL and DL-trained cohorts. Specifically, we attributed the expected difference to the DVL cohorts’ ability to directly visualize the anatomy and simultaneously receive instructor feedback via a real-time video feed. To test this hypothesis, we designed a study using medical student participants to investigate the efficacy and impact of three standardized training procedures, DL, IVL, and DVL, in a manikin-based simulation laboratory on the student's proficiency and confidence in performing DL. To our knowledge, this is the first study to directly compare all three modalities simultaneously.

## Materials and methods

Participant recruitment and consent

The research project #2022-062 was granted exemption by the Edward Via College of Osteopathic Medicine, Virginia Campus, Institutional Review Board. Following determination, participants were recruited and consented.

Participants were recruited via email from the Edward Via College of Osteopathic Medicine, Virginia Campus, as a convenience sample. Multiple emails were distributed to first-year medical students for recruitment. Additionally, in-person recruitment talks were held during the first five minutes of a class period to recruit and answer questions regarding the study. Following recruitment, a consent form was emailed to those who volunteered to participate. Researchers’ contact information was provided in the email sent with the consent form for questions to be answered regarding the study. After consent forms were returned, the team contacted each participant via email to schedule their pre-assessment. In total, 32 participants were recruited for the study; one withdrew prior to completion.

Study design

Participants were assigned unique identification numbers using Microsoft Excel (Microsoft Corp., Redmond, WA) and randomly allocated to one of the three groups - DL, IVL, or DVL - prior to undergoing their pre-assessment. This process involved data entry, deletion, and secure recording of ID numbers to ensure participant confidentiality. During their pre-assessment phase, participants were isolated in a private room and shown an overview video on intubation protocol before engaging in the pre-assessment evaluation of DL on a manikin. Following completion of the pre-assessment, participants were scheduled and underwent training in their designated cohorts. During their training, participants were trained with other participants within their designated cohort. Participants were then scheduled following their training for a post-assessment. During this phase, participants were again isolated as they were in their pre-assessment and evaluated via performing DL on a manikin. Metrics for both pre- and post-training assessments included successful intubation, time to successful intubation, NRS to gauge trainee self-perception and confidence level in performing intubation in a future real-life scenario, and dental injury occurrence as this is a common injury that occurs during intubation [14,15].

Pre-assessment

A pre-assessment was utilized to gather a participant baseline, thereby establishing participants’ pre-intervention knowledge for post-assessment comparison. In total, 32 participants were recruited for the study; one withdrew prior to completion. Subsequently, participants were individually escorted to a designated room where the consent form was reviewed, and any additional questions were answered.

Next, participants completed a demographics survey covering gender, age, ethnicity, healthcare experience, prior intubation education exposure, and both prior and current video game experience. Participants then viewed a brief instructional video on intubation protocol before participating in the pre-assessment. The pre-assessment, conducted individually, measured successful intubation, time to successful intubation, and dental injury. Participants self-reported their confidence levels and perceptions of performance through an NRS, respectively. The NRS was presented on a blank page as a freely moveable linear slider void of any numerical markings. The left side of the slider demonstrated a red hue, signifying a less confident rating, and the right side emitted a green hue, demonstrating a more confident rating. Internally, the NRS provided researchers with a numerical equivalent starting at zero on the left side and ending with 100 on the right side. Participants were permitted five minutes to complete their intubation attempt, beginning at the passage of the laryngoscope through the lip line of the manikin.

Cohort training

Following the pre-assessment, researchers scheduled participants for training. Each cohort received a one-hour training led by a medical student instructor. The instructor, having completed intubation training and assessment at the Edward Via College of Osteopathic Medicine and an additional competency check-off from a board-certified emergency medicine physician, delivered a standardized presentation on equipment, indications, contraindications, and basic DL techniques for the first 10 minutes. The remaining time in the session focused on kinesthetic learning tailored to the specific group assignment, either DL, IVL, or DVL.

**Figure 1:**
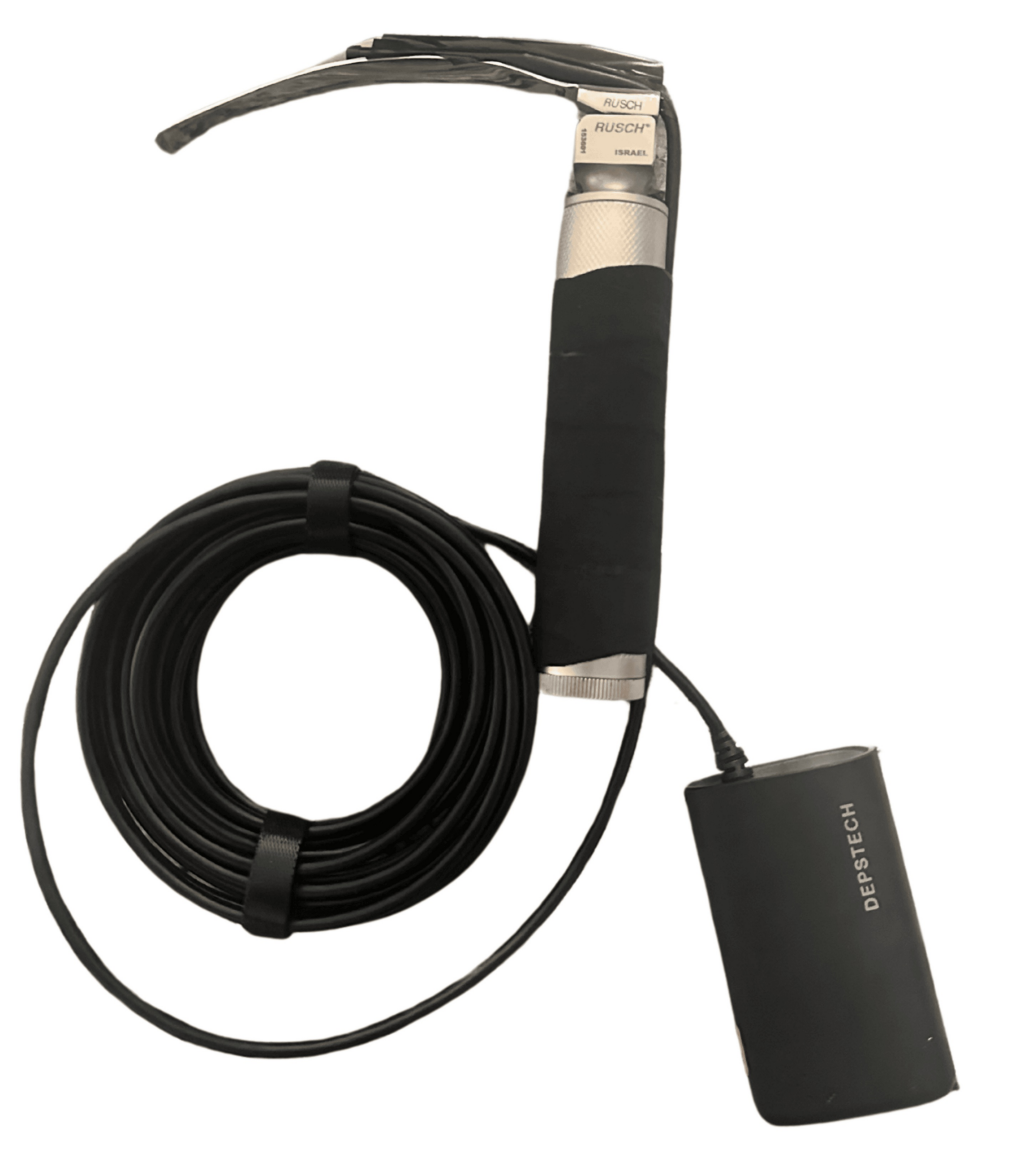
Direct Video Laryngoscopy training device.

**Figure 2:**
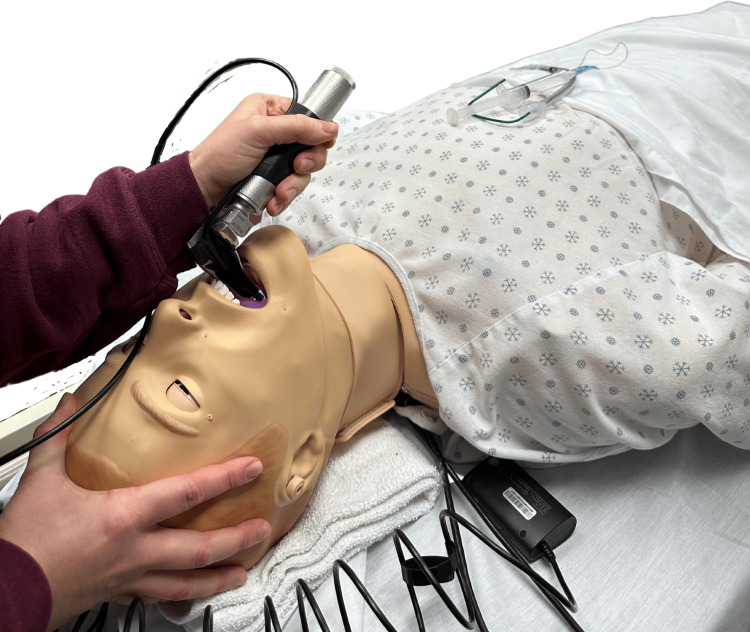
Demonstration of the Direct Video Laryngoscopy training device on the manikin.

**Figure 3:**
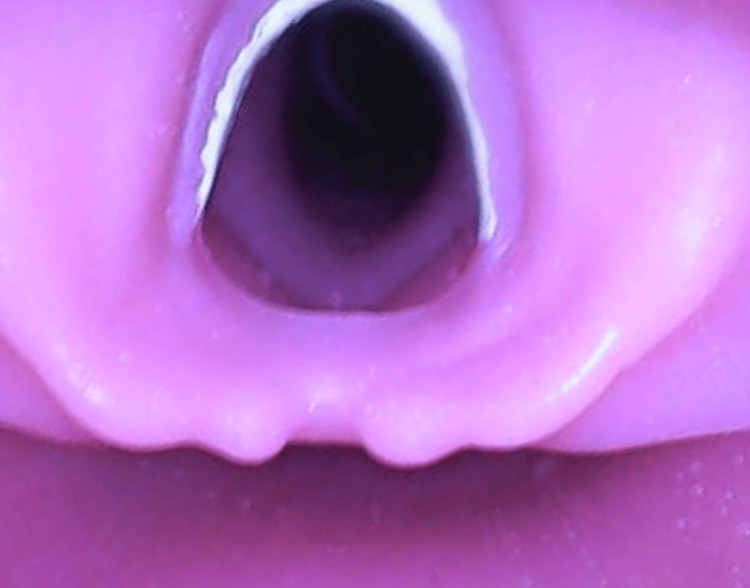
Demonstration of the view the instructor has for providing feedback during Direct Video Laryngoscopy.

DL participants visualized anatomy using a size 3 Macintosh blade (Medtronic, Minneapolis, MN), with the instructor blinded to the anatomy, relying solely on verbal feedback for guidance. The DVL group trained via a hybrid tool, using the same laryngoscope used in DL with a camera mounted to the blade (Figure 1 and Figure 2). This allowed participants to direct visualization while giving the instructor indirect or video visualization to provide feedback (Figure 3). IVL participants used a GlideScope® Titanium Reusable model (Verathon, Bothell, WA) with the GlideScope® Core monitor (Verathon, Bothell, WA) for indirect anatomy visualization. The instructor provided real-time guidance by viewing the same GlideScope® monitor as the participant.

Post-assessment and training sessions

Following the training sessions, participants were scheduled for their post-assessment. Participants performed intubation with DL, and the same data points were collected as in the pre-assessment. This included achievement of successful intubation, time to successful intubation, dental injury, NRS to assess the trainee’s perception of their performance, and confidence level of performing intubation in a real-life scenario.

Successful intubation, time, and dental injury were recorded during the procedure. Video recordings were reviewed for result validity if necessary. All data were organized by number identifiers into pre- and post-categories.

Airway assessments

Manikin-based assessments and training were carried out using the Laerdal platform (Laerdal Medical, Stavanger, Norway), including high-fidelity manikins such as SimMom, SimMan 3G Plus, SimMan 3G, and SimMan Essentials (all are manufactured by Laerdal Medical, Stavanger, Norway). Participants were randomly assigned to manikins for pre- and post-assessments. All Laerdal manikins were operated and connected through the Laerdal Learning Application (LLEAP) software (Laerdal Medical, Stavanger, Norway). Cohorts were evaluated based on successful intubation, time to successful intubation, dental injury, and NRS to assess the trainee’s perception of their performance and confidence level of performing intubation in a real-life scenario.

Successful intubation, for the purposes of this study, is defined as the placement of the endotracheal tube in the trachea within the five-minute time limit. Participants were given a total of five minutes for their assessment. This included equipment setup, for example, attachment of the laryngoscope blade to the handle, passing the laryngoscope blade through the lip-line of the manikin, attaching the bag-valve mask, and performing the first ventilation with bilateral lung inflation as registered by the LLEAP software. The LLEAP software demonstrated an “assisted ventilation” notification at the first and all subsequent detections of externally supplied ventilation, i.e., via bag-valve mask. Importantly, misplacement of the endotracheal tube into a mainstem bronchus would only produce a unilateral notification of “assisted ventilation,” while esophageal intubations did not produce a notification. Time to successful intubation was derived similarly, as the total duration from the first time the laryngoscope passed the lip-line to detection of successful ventilation as previously described. Participants' time continued even if they removed the equipment from the mouth for a second pass. Exceeding the maximum allocated time, meaning greater than five minutes (300 seconds), or failure to successfully intubate, resulted in the exclusion of the participant from the final analysis of time to successful intubation; however, their data were analyzed for the remaining study metrics. These participants were evaluated in dental injury and both NRS confidence and self-perception, as we had a small sample size and believed these variables were important to evaluate individually. Investigators visually noted dental injury during assessments if a participant deviated from the standard DL technique, specifically looking for any retroflexion of the laryngoscope or placement of the blade directly onto the teeth. This was observed during the encounter and verified, if necessary, via video review.

Data analysis

Statistical analysis, including ANOVA, Pearson's chi-squared test, Fisher's exact, Friedman rank sum test, and the Benjamini-Hochberg method, was employed to analyze pre- and post-assessment data. The study aimed to comprehensively evaluate the effectiveness of different intubation education modalities and their influence on participant performance and confidence levels. Data analysis focused on 21 participants (n = 21), excluding participants with prior intubation experience and one withdrawn participant, out of the 32 participants initially recruited. Time to successful intubation was only evaluated post-assessment as only one participant successfully intubated during the pre-assessment.

## Results

**Figure 4:**
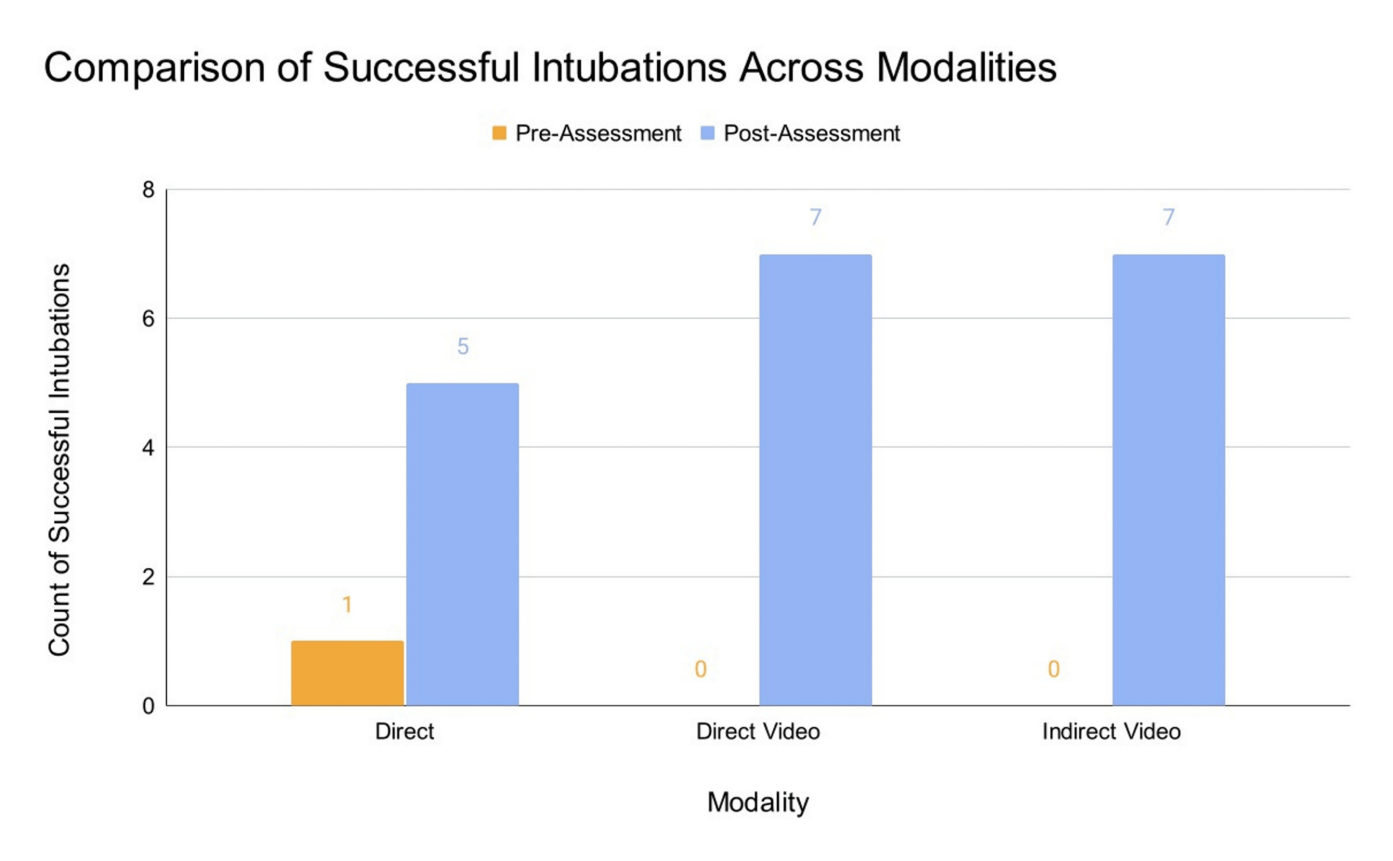
Comparison of successful intubation counts across modalities pre and post-assessment. n = 7 for each modality. None of the participants in the DVL and IVL groups successfully completed the intubation pre-assessment. DVL: Direct Video Laryngoscopy, IVL: Indirect Video Laryngoscopy

**Figure 5:**
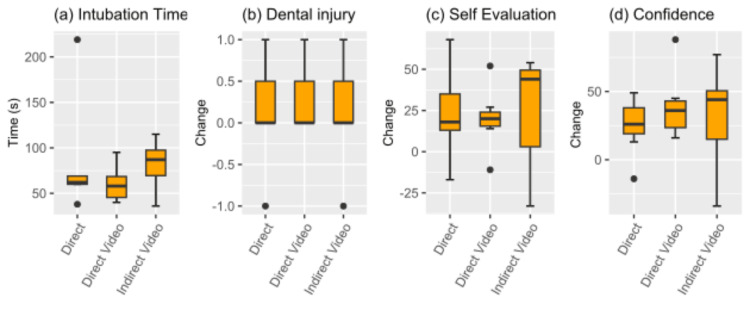
Comparison of DL proficiency. n = 7 for each of the three modalities. (a) Box plots of post-assessment times to intubate are depicted. (b-d) The box plots represent changes observed between pre- and post-assessments across various metrics. Positive values indicate improvement, negative values indicate deterioration, and zero indicates no change between pre- and post-assessments. (b) A positive change was seen across all three modalities, indicating a decrease in dental injuries post-assessment. However, the Friedman test yielded a p-value of 0.93, suggesting no significant difference. (c) Average differences between pre- and post-assessment NRS responses to the question "How did you feel you performed in this simulation?" are shown. The p-value for the Friedman test was 0.65, indicating no significant difference. A positive change value suggests an improvement in participants' self-rating compared to pre-assessment. (d) Average differences between pre- and post-assessment NRS responses to the question "How confident do you feel in your ability to perform a real-world intubation?" are illustrated. The p-value for the Friedman test was 0.37, indicating no significant difference in the observed differences. A positive change value suggests an improvement in participants' self-rating compared to pre-assessment. DL: Direct Laryngoscopy, NRS: Numeric Rating Scale

Demographics analysis revealed a predominantly female participant group (67%), the majority falling within the 21-25 age bracket (95%) (Table 1). Statistical evaluation of demographics and outcomes utilized ANOVA, Friedman rank sum, and Fisher's exact; results revealed the median changes in self-perception and confidence were significantly different. The p-values were corrected for multiple comparisons using the Benjamini-Hochberg method (adjusted p-value < 0.05). Analysis of self-perception and demographics revealed statistical significance in the following: age (p = 0.001), ethnicity (p = 0.004), prior intubation exposure (p = 0.004), and current video game time per week (p = 0.004). Analysis of confidence in performing intubation in a real-life scenario and demographics revealed statistical significance in the following: age (p = 0.001), ethnicity (p = 0.004), prior intubation exposure (p = 0.003), and current video game time per week (p = 0.001). No statistical significance was observed in the following demographic categories: prior healthcare experience, gender, or prior video game experience. Assessment of outcomes based on modality, employing ANOVA, Pearson's chi-squared test, and the Friedman rank sum test, yielded no statistical significance. For a power of 80%, the sample size was calculated as 162 participants, with 54 participants per cohort. While IVL and DVL cohorts achieved 100% success following training, compared to 71% in the DL cohort, the results were not statistically significant, as demonstrated by Pearson's chi-squared test (p = 0.1096) (Figure 4). A positive change in dental injury, self-evaluation, and confidence metrics was seen across all three modalities in the post-assessment analysis (Figure 5). However, the difference between training modalities was not found to be statistically significant. The one-way ANOVA test did not demonstrate statistical significance for time to successful intubation (p = 0.459). The Friedman rank sum test did not demonstrate statistical significance for dental injury (p = 0.926), self-perception of performance (p = 0.6514), or confidence in performing intubation in a real-life scenario (p = 0.3679).

**Table 1:** Demographics data of participants in this study (n = 21) All percentages reported with respect to n = 7, per cohort.

Variable	Direct (n = 7)	Direct Video (n = 7)	Indirect Video (n = 7)
Gender			
Male	2 (28.6%)	3 (42.9%)	2 (28.6%)
Female	5 (71.4%)	4 (57.1%)	5 (71.4%)
Age (years)			
21-25	7 (100%)	6 (85.7%)	7 (100%)
26-30	0 (0%)	1 (14.3%)	0 (0%)
Ethnicity			
Asian	4 (57.1%)	2 (28.6%)	1 (14.3%)
Caucasian	3 (42.9%)	4 (57.1%)	5 (71.4%)
Latino or Hispanic	0 (0%)	1 (14.3%)	0 (0%)
Middle Eastern	0 (0%)	0 (0%)	1 (14.3%)
Do you have any previous experience in the healthcare field (select all that apply)?			
Urgent care	2 (28.6%)	0 (0%)	0 (0%)
Emergency medical services	2 (28.6%)	0 (0%)	1 (14.3%)
Hospital based	1 (14.3%)	0 (0%)	2 (28.6%)
Outpatient	3 (42.9%)	4 (57.1%)	1 (14.3%)
Pharmacy technician	0 (0%)	1 (14.3%)	0 (0%)
Scribe	0 (0%)	0 (0%)	2 (28.6%)
None	1 (14.3%)	2 (28.6%)	2 (28.6%)
Previous intubation experience?			
Lecture-based learning	1 (14.3%)	0 (0%)	1 (14.3%)
None	6 (85.7%)	7 (100%)	6 (85.7%)
In the past, how many hours per week did you play video games (hours)?			
None	2 (28.6%)	1 (14.3%)	0 (0%)
0-3	4 (57.1%)	3 (42.9%)	4 (57.1%)
4-7	0 (0%)	2 (28.6%)	3 (42.9%)
8-11	1 (14.3%)	1 (14.3%)	0 (0%)
Currently, how many hours per week do you play video games (hours)?			
None	2 (28.6%)	1 (14.3%)	0 (0%)
0-3	5 (71.4%)	6 (85.7%)	5 (71.4%)
4-7	0 (0%)	0 (0%)	2 (28.6%)

## Discussion

Our study evaluated the efficacy of three common intubation education modalities across five metrics. To our knowledge, this study is the first to compare all three methods simultaneously. The metrics chosen are of particular importance due to their implication in real-life scenarios. Although participants were trained with different modalities, they were consistently evaluated with DL. A 2017 study performed in the United Kingdom found VL availability was highest in main operating departments, but less than half of other locations, including the intensive care unit, emergency department, and obstetrics theaters, had VL available [16]. Our goal was to evaluate participants trained with varying modalities and their ability to utilize DL for real-world application, as physicians are likely to encounter a situation in which they will not have access to VL, requiring immediate DL to secure an airway. Furthermore, previous research has demonstrated the ability of participants to be trained with VL with sufficient or improved results when evaluated with DL [17,18]. An individual’s ability to quickly and accurately perform an intubation is a crucial, life-saving technique. In evaluating the time needed for successful intubation, we aimed to capture this importance. Our third metric, dental injury, was key for evaluation, as it is a common complication in intubation [14,15]. Techniques to mitigate undue pressure on teeth are essential, though injury may still occur due to other factors, including dental conditions [19]. The last two metrics evaluated the perception of the participant. Through the utilization of an NRS, we evaluated how the participant viewed their performance, as well as how confident they felt performing intubation in a real-life scenario. The metrics encapsulate the various important aspects of ETI education. Analyzing different aspects of training, including quantitative measurements of time, success, and injury, coupled with qualitative aspects of how participants felt following training, is vital for a well-rounded, confident, and competent practice. 

Herbstreit et al., in 2011, demonstrated the superiority of DVL compared to DL in the evaluation of intubation success and time to intubation [20]. Research by Yi et al. in 2021 exemplified the superiority of DVL compared to IVL in the evaluation of time to intubation [9]. Comparatively, our study failed to demonstrate the statistical superiority of any training cohort in the evaluation of DL. Our limited sample size of 21 out of the estimated 162 participants, needed to satisfy our power calculation, likely inhibited our ability to elucidate a difference in the modality of intubation education. The generalizability of this study is limited, as all participants were currently enrolled in medical school. However, it is important to consider the general paucity of literature pertaining to laryngoscopic education in the medical student population. Our study demonstrated statistical significance in the evaluation of demographics data, including age, ethnicity, prior intubation exposure, and current weekly video game experience; however, we are hesitant to place much value on these findings given the limited sample size and, thus, limited representation of various demographic profiles. Namely, only one participant was in the 26- to 30-year-old age group, one participant was Latino, two participants had lecture-based previous exposure, and two currently played four to seven hours of video games per week. Furthermore, we acknowledge the rudimentary evaluation of dental injury we employed in this study; future implementations may involve the implantation of pressure or force sensors along the crowns of the teeth. An attempt should be made to standardize the utilization of the same manikins for pre- and post-assessment intubations to limit this potentially confounding variable. Lastly, future studies should consider a primary metric of first-pass intubation rate in addition to successful intubation within a given time.

## Conclusions

The observations made from this study warrant further investigation with a larger sample size and encourage refinement of the modalities with which we record objective assessment data.
